# Exploring the communication between telenurse and caller—A critical discourse analysis

**DOI:** 10.3402/qhw.v9.24255

**Published:** 2014-06-24

**Authors:** Roya Hakimnia, Inger K. Holmström, Marianne Carlsson, Anna T. Höglund

**Affiliations:** Department of Public Health and Caring Sciences, Uppsala, Sweden

**Keywords:** Authentic calls, communication, discourse analysis, gender, telenursing, Sweden

## Abstract

**Background:**

Telenursing is an expanding service in most Western societies. Sweden is a front-line country, with all of its 21 counties connected to Swedish Healthcare Direct (SHD) 1177. The intention of the service is twofold: to make health care more efficient, while also making it more accessible and safe for patients. Previous research has shown, however, that the service is not used equitably. Gender, age, socio-economic, and ethnicity differences have been reported as determining factors for the use of the service and the advice given.

**Aim:**

The aim of the study was to explore the communication between telenurses and callers in authentic calls to SHD 1177.

**Methodology:**

A qualitative method, using critical discourse analysis (CDA), was chosen. The approach was deductive, that is, the analysis was made in view of a predetermined framework of theory. Twenty calls were strategically chosen and included in the study.

**Results:**

The CDA resulted in five types of calls, namely a gatekeeping call, a gendered call, a call marked by impersonal traits, a call with voices of the life world, and finally a counter discourse call. The dominating patterns in the calls were of gatekeeping and biomedical character. Patterns of the societal gender order were found, in that representations of the reluctant male caller and the ideal female caller were identified, but also a call representing a counter discourse. The service seemed difficult to use for patients with low language proficiency.

**Conclusion:**

Telenursing could potentially challenge inequalities in health care. However, the discourse of telenursing is dialectically related to neoliberal ideology and the ideology of medicine. It is also situated in a gendered context of ideal femininity and hegemonic masculinity. Through better awareness of gender biases and the callers’ different resources for making themselves heard, the communication between telenurse and caller might become more equal and thereby better suitable for all callers.

Telenursing is an expanding service in many countries, for example, the United Kingdom, Australia, and the United States (Collett, Kent, & Swain, 2006). Sweden is a front-line society, with all of its 21 counties connected to Swedish Healthcare Direct (SHD) 1177. The intention of the service is twofold: to make health care more efficient while also making it more accessible and safe for patients (www.1177.se).

The telenurse's task is to direct the caller to the right level of health care or give self-care advice. A telenurse in Sweden handles six to eight calls per hour (Swedin, 2003). Assessing, referring, and giving advice, but also being supportive and teaching the caller, are important aspects of telenursing (Kaminsky, Carlsson, Höglund, & Holmström, 2010; Swedin, 2003). Telenurses have expressed both satisfaction with their work and concern about the monotony of working at a call-centre-like milieu (Knowles, O'Cathain, Morrell, Munro, & Nicholl, 2002). The telenurse is supposed to be both a professional caregiver and a gatekeeper, which has been reported to occasionally create dilemmas (Holmström & Dall'Alba, 2002). Swedish telenurses are obliged to use a computerized decision software system, CDSS, which telenurses have described as both supportive and as a hindrance to autonomy (Ernesäter, Holmström, & Engström, 2009).

Swedish health care is supposed to be distributed equally and with respect for human dignity (SFS 1982:763). Traditionally, the system has been based on tax. For the past 20 years, Swedish health care has undergone key changes within the neoliberal realm, with New Public Management (NPM) reforms and privatization at its centre (Fredriksson, 2012; Jonvallen, Berg, & Barry, 2011). The idea behind NPM is to transfer ideals and methods from the private to the public sphere. Still, no more than 10% of the Swedish care providers are private and few patients have private health care insurances (svenskforsakring.se/en).

Telenursing is a service inspired by call centres at private companies (Andersson Bäck, 2008) and has been seen as a cost-effective way to provide health care (Jennett et al., 2003). However, previous studies indicate that ethnic minorities, deprived groups, and the elderly tend to underuse the service (Cooper, Arnold, Smith, Hollyoak, & Chinemana, 2005; Hsu, Bath, Large, & Williams, 2011; Knowles, Munro, O'Cathain, & Nicholl, 2006; Shah & Cook, 2008; Waqas, Theivendra, Sood, Vasireddy, & Maryon-Davis, 2003). Telenursing has also been described as a gendered service, in that it is both handled and used mainly by women (Höglund & Holmström, 2008). Different types of male callers have been identified: the assertive carer, the reluctant patient, and the new dad (Goode et al., 2004). Telenurses have experienced male callers as either assertive or reluctant, whereas they described female callers as easier to persuade to wait and see (Höglund & Holmström, 2008). Differences have also been reported concerning the advice given by telenurses. A Swedish study showed that the likelihood for a father to receive referral to health services from a telenurse were twice as great as for mothers (Kaminsky et al., 2010).

## Theoretical framework

Gender relates to the social constructions of femininity and masculinity and has been described as something active; we are continuously “doing gender” (West & Zimmerman, 1987). The gender order is a relational and contextual structure, which in most societies subordinate femininity in relation to hegemonic masculinity (Connell & Messerschmidt, 2005). “Doing health” has been shown to be an important aspect of “doing gender” (Saltonstall, 1993); for example, hegemonic masculinity can be reinforced by men being reluctant to seek health care, taking risks in traffic, or drinking excessively (Addis & Mahalik, 2003; Courtenay, 2000; Noone & Stephens, 2008; Schofield, Connell, Walker, Wood, & Butland, 2000). At the same time, ideal femininity is upheld by the opposite health behaviour, that is, taking care of one's own and other family members’ health and seeking health care in time (Lyons, 2009). However, in accordance with unequal gender-related power, it has been found that women often have to wait longer for treatment for various medical conditions (Smirthwaite, 2007).

Gender also interacts, or intersects, with other social categories, such as age, socio-economic position, sexuality, and ethnicity (Weber & Parrah-Medina, 2003). This has also been reported concerning health behaviour (Hankivsky, 2012). Wamala, Merlo, and Boström (2007) found that perceived discrimination due to more than one social category increased the risk of refraining from seeking health care up to nine times.

These results indicate that health care is not always distributed according to principles of justice. Justice can be seen both as a distributive concept (focused upon how the goods of a society should be divided) and as a retributive concept (focusing on just treatment or punishment according to deeds). In health care, mainly justice as distribution is relevant, as it concerns the distribution of social goods in a society. There are different understandings of what constitutes as just or fair distribution of social goods. A well-established position is Rawls’ (1972) theory which prescribes that the distribution should be equal, unless an unequal distribution is in the favour of the least privileged groups in society. Michael Walzer (1983) argues in *Spheres of Justice* that the possession of one type of social good, for example, money, should not automatically give access to other social goods, such as health care. Previous studies (Cooper et al., 2005; Hsu et al., 2011; Knowles et al., 2006; Shah & Cook, 2008; Waqas et al., 2003) indicate that ethnicity, class, and gender, that is, factors related to power, position, and influence in a society do give favours also when it comes to health care; a matter that will be critically discussed in the following.

## Aim

The aim of this study was to explore the communication between telenurses and callers in authentic calls to SHD 1177.

## Methodology

The study was performed with qualitative methodology, using critical discourse analysis (CDA). CDA sees discourse as the use of language in speech and writing as a form of social practice. The basic interest for all critical discourse analysts is power and social change (Van Dijk, 1993). Fairclough (1992) has suggested a three-dimensional framework for conceiving discourse analysis. The first dimension is *text analysis*. At this level, the researcher is interested in the micro-aspects of discourse practice and focus is on choices of patterns in vocabulary, grammar, cohesion, and text structure. In the present study, the “text” will be telephone calls to SHD. The second dimension is *discursive practices*, which means understanding discourse as something that is produced, circulated, distributed, and consumed in society. The third dimension understands discourse as a *social practice* with ideological effects and sees it as part of hegemonic processes.

Mishler (1984) has argued that the medical discourse is divided into two separate and dialectical discourses whereby the patient expresses *the voice of life world*, whereas the doctor talks in *the voice of medicine*, oriented almost exclusively towards a technical bioscientific understanding of medicine (Mishler, 1984). Waitzkin (1989, 2011) further points out that the medical meeting does not occur in a vacuum; many problems the patient brings to the doctor have societal roots—thus micro-level interactions are shaped by macro-structures of society. Alongside the educational system and mass media, medicine is an institution that instils dominant ideologies in the population (Waitzkin, 1989). Hence, discourse is both socially constitutive and socially conditioned. Translated to the context of telenursing, health calls both reflect and shape the societal discourse. In this study, CDA was chosen as methodology so that power interactions in the calls would be possible to identify.

### Setting

The study was conducted in 2011–2012 in a medium-sized city in Sweden in a 24 h per day reachable telenursing site. The telenurses at the site assessed symptoms, gave advice or referrals, and booked appointments for GPs on call.

### Data collection

All 31 telenurses at the site were asked to participate. Of these, 11 agreed to take part; 10 women and 1 man. In order to ensure confidentiality, all telenurses are in the following referred to as “she.” For 1 week, calls to this telenursing site were collected. From approximately 1000 calls, 20 were strategically chosen. The number of 20 was considered suitable for the CDA, that is, big enough to give a variation in the results but not so big that the data would be difficult to handle. Inclusion criteria were: 1) the caller was calling for her- or himself; 2) the caller was 18 years of age or older; and 3) the caller was calling for a medical condition. Of the 20 chosen calls, 13 were made by women and 7 by men.

### Analysis

The calls were transcribed verbatim. The transcripts were read through several times to locate moments of crisis, when the “normal” is interrupted and dominance relations are revealed. Following Fairclough (1992), Mishler (1984), and Waitzkin (1989), the following questions were studied in the text:

*What turn-taking rules are in operation and who makes use of them?*

*Who holds the topic control and how is it maintained?*

*How and by whom is the agenda set?*

*What are the characteristics of the questions and responses?*

*What are the power relations in the call?*

*Are the voices of life world and voices of medicine voiced harmonically?*

*Is ideological work performed?*

*Are social categories, such as gender, age, ethnicity, and socioeconomics, at work?*

The approach was deductive, in that the analysis was made in view of a given structure and predetermined framework of theory (Burnard, Gill, Stewart, Treasure, & Chadwick, 2008). A first analysis was made by the first author (RH). Thereafter, the other authors read transcripts and participated in the analysis of the calls. The final version of the result presentation was approved in consensus with all authors.

### Ethical considerations

The study concerned the encounter between telenurse and caller, with focus on power interactions related to factors such as gender and ethnicity. Hence, the issue could be regarded as sensitive for the participating nurses and callers. In order to handle these ethical challenges, much effort was put on information and the consent process. Two of the participating researchers (RH and ATH) informed the telenurses verbally in a workplace meeting, where the nurses had the opportunity to ask questions and get clarifications. Thereafter, written information on the study was handed out. The information emphasized that participation was voluntary and it was possible to withdraw at any time. Likewise, it was guaranteed that data should be handled confidentially and that the workplace would not be identifiable. The nurses consented to participate through signing a written consent form. The callers were informed about the study through a pre-recorded message and consented to participate by pressing a button on the telephone. Also in this information, it was stressed that participation was voluntary and that all participants’ confidentiality was guaranteed. The study was approved by the regional ethics review board, DNR 2012/156.

## Results

Through the CDA, five types of calls were identified, namely a gatekeeping call; a gendered call; a call marked by impersonal traits; a call with voices of the life world; and, finally, a call expressing a counter discourse. In the following, each type of call has been illustrated by transcripts. In the transcripts, T stands for “telenurse” and C for “caller.” A full stop (.) represents a one-second pause, a comma (,) a half-second pause. Capital letters signal intonation. Parentheses enclose descriptions and not transcribed utterances.

### Call number 1: A gatekeeping call

In the first call, an elderly male patient is having difficulty describing his problem in an adequate medical language.

C: Eh … Well, I have some problems with … yes … eh with my knee; I don't know what's happened to it.

T: Ah really? (Sounds surprised)

C: Yeah, one of my knees has been aching and I've been to the primary care centre and got a shot in it but … now it's moved to the other knee.

T: You don't say? (Sounds surprised)

C: Yeah … it has …

T: Is it the same … the same kind of ache?

C: No it's worse. I called the primary care centre but …. they didn't have any appointments until Wednesday and they told me to call 1177. I … I don't know where I should go so … well ….

T: Yeah, well (sighs) … I guess it's not … Did you get an appointment on Wednesday, then?

C: No, no, I didn't, I don't know what you can do in these cases, I guess there's not much you can do …

T: No there isn't, so I think you kind of should've taken that appointment, you know … on Wednesday.

The caller seems unsure and running the risk of getting lost in the shuffle, as primary health care urges him to call 1177, and the telenurse tells him to call primary health care back. His lack of medical words and little knowledge about the health care organization contrasts with the telenurse's knowledge, and she can easily steer him. The power asymmetry could have been challenged by, for example, more questions about the knee or other symptoms, but this was not done. The gatekeeping role takes over and the focus becomes on directing the man back to the GP. There are few elements of nursing in the call; few supportive or caring words are expressed; and it is first and foremost an efficient call.

### Call number 2: A gendered call

In the second call, a young girl complains about her cough. The telenurse listens to her and becomes immediately engaged.

T: Well I REALLY think somebody should listen to your lungs

C: Yeah … and I called them and said that I really, really wanted that but they said they didn't have any appointments today, but that I should call 1177 (expresses criticism).

T: Well, I could get you an appointment at the GP on-call centre.

C: Mm, THAT would be GREAT (expresses approval).

T: Then you'll … you'll get another doctor too … if I understand it correctly … but this could be good if you've already asked for help and they haven't listened.

C: Yes, exactly (expresses approval).

(Nurse types on her computer)

T: Let's see how it is with appointments, ‘cos you surely can't walk around like this anymore (with empathy).

C: No, I can't even SLEEP (sounds concerned).

T: No, no, that's bad … but we'll manage this together, so it'll be okay (with empathy).

Here, the telenurse shows empathy and takes the caller's side. She builds a partnership, both with the health care system in general and with the caller, using words like “we.” The nurse almost acts like a caring mother of this young female caller, in contrast to the first call from an elderly male caller in which the communication was more direct and technical. Both callers express worries, but are encountered differently. The young girl's help-seeking behaviour is approved, whereas the reluctant male caller's help-seeking behaviour is not.

### Call number 3: An impersonal call

In the next example, a male immigrant, who has difficulty describing his problems because of low Swedish proficiency, calls in to the service.

C: Well, it is like this … that I got some ache in my throat and I can feel it all up to my ear, you know.

T: Mm, when did your throat start to hurt?

C: It is just a day ago … (difficult to understand patient's language)

T: Mm …

C: It hurts all the way to the ear, you know.

T: Mm …

C: I have asthma and I have only 30 percent of my kidney capacity left.

T: Mm …

C: So I don't know, I am a bit afraid that it is an infection, so that is why I wanted to come to the … ehh … the GP on call …

So far the call is short and sounds mechanical, and lacks both medical questions and social exchange. Because the caller is not proficient in Swedish, he needs to be clear about what he needs, and asking for service is one of the few power tools he as caller possesses. This power is quite weak, however, as it is still the telenurse who decides. In the following, the man answers the telenurse's strictly medical questions, but also tries to make the call a bit personal at one point by trying to make a joke.

T: What did you say, your kidneys have low capacity?

C: Yes, I am a diabetic and I have around 30% of my function left and I have high blood pressure and I have asthma too … I have everything haha … (laughter)

(Nurse typing on the computer, quiet for six seconds)

T: Let's see then, do you have any fever today?

Here, the caller shows a glimpse of his life world but the telenurse returns back to the voice of medicine. This creates a certain power asymmetry, as the nurse can easily go back to the mechanical and medical talk after the patient's joke. The call ends with the telenurse giving the man an appointment. This call is also gatekeeping in its character because there are few questions about the symptoms or the caller's experience of them.

### Call number 4: A call with voices of the life world

The caller in this example is a young girl whose symptoms are neurological. At one point, the woman explains that “it feels like I'm not in my body” and the nurse tries to understand this life world expression through her medical language.

T: But you don't have any … you don't have any feelings of numbness or focal deficits otherwise?

C: No, but I feel a bit weird … it feels as if … like when I'm walking … or like yesterday for example it felt as if I couldn't feel the lower part of my body … or my arm but not from the shoulder down to … ehm … the elbow.

T: Mm … (Nurse typing on her computer)

C: Then it felt like … yeah, it's very difficult to explain but I … I felt like from my elbow down to my … fingers, but not up to … in a strange way …

A child cries in the background and the woman explains that she has recently delivered and that she has been very stressed out. The telenurse answers that stress can be the cause of the symptoms. However, no questions about the root of the stress are posed, for example, concerning the new experience of being a mother or about housework division. Here a clash between the caller's voice of life world and the telenurse's voice of medicine occurs. Another clash occurs when the computerized decision program suggests that the young woman needs to go to the emergency room, but the telenurse hesitates.

T: But … in my support program it says that if you haven't been assessed you should go and get examined for these symptoms.

C: Okay …. Yeah, I'll call … my … what's it called … general practitioner.

T: Well, in my advice it says you have to go immediately.

C: Aha, okay.

The call ends and the caller is left with two contradictory pieces of advice; that the symptoms are temporary and due to stress, or that she needs to go to the emergency department immediately.

### Call number 5: A counter discourse call

In the final example, the caller is a man who calls about coughing and feeling tired.

T: So you're tired but not very tired? (Expresses empathy)

C: Exactly, I don't know if I need more sleep since I'm not doing anything … or whatever's happening.

T: Mm … mm … But how's your breathing? Are you still breathing normally?

C: Yeah, I think so. I don't cough very much, not so much that it tears in my chest when I cough, but … I don't cough frenetically but it rather comes in fits and then I cough but it's over in a moment, kind of.

T: Mm …

In the beginning of the call, the telenurse acknowledges the man's life world by repeating his own words (“tired, but not very tired”). Thereby, this call challenges the dominant discourse of telenursing; the medical approach is broadened and nursing elements are present. The telenurse takes the caller's medical history seriously, asking about medications and allergies, and about other symptoms, and even asks him to examine himself.

T: That's good, and you're healthy otherwise or do you have any other diseases?

C: No, I'm healthy.

T: Do you take any medications on a regular basis?

C: No.

T: Do you have any allergies?

C: No.

T: Yes, and I want to ask you, can you tilt down your chin toward your chest?

C: … … Yeah, I can.

T: That's fine.

At the end of the call, the telenurse also explains why she gives different pieces of advice.

T: Mmm … and … eh … you can also treat this by drinking a lot so the mucus gets thinner and it becomes easier for the body to get rid of it … It decreases the risk of pneumonia, and another thing you can do is to change your position all the time so you get your mucus to loosen up.

The power in the call is mainly in the hands of the telenurse. She talks more than the caller, but analyses his symptoms thoroughly and explains her advice, teaching him more about his own body. This challenges the knowledge asymmetry between them. Still, the call mainly concerns medical questions and the nurse talks in the voice of medicine, but with a nursing touch, and with the allowance of life world expressions to take part in the conversation. Thereby, it is possible to identify this call as a counter discourse.

## Discussion

The aim of this study was to explore the communication between telenurse and caller in authentic calls to SHD 1177. CDA was chosen as methodology, so that power interactions in the calls would be possible to identify. The CDA revealed how the voice of medicine and a gatekeeping character dominated the calls. Patterns of the societal gender order were found, but also one example of a counter discourse. Five types of calls were identified, namely *a gatekeeping call*, *a gendered call*, *a call marked by impersonal traits*, *a call marked by the voices of the life world*, and finally, *a call expressing a counter discourse*.

The five types of calls can be grouped together in three wider themes, namely a *gatekeeping theme*, *a medicine focused theme*, and a theme concerning *doing gender*. The counter discourse that was found resisted these themes, which is the function of a counter discourse, but at the same time it can contribute to the constituting of the other discourses as hegemonic.

In *gatekeeping calls*, the telenurse primarily directs the caller to the right level of health care, at the expense of advice and emotional support. This was found in call number 1 and call number 3. This type of communication seems to harmonize with one goal of telenursing, namely to prevent unnecessary health care visits (Andersson Bäck, 2008; Swedin, 2003). On the other hand, it clashes with other goals in telenursing, which are to support the callers and meet their worries (Swedin, 2003). Traditional elements of nursing, such as showing empathy and using open questions to learn about the patient's unique understanding of his or her symptoms (Travelbee, 1971), are rarely found in gatekeeping calls. Furthermore, the “laying on of hands,” that is, the intimate touch that distinguishes medical encounters from other professional–client interactions (Waitzkin, 1989), is absent in telenursing; an aspect that might contribute to the gatekeeping role.

Telenursing seems to have one foot in neoliberal efficiency ideologies and one in the ideologies of increasing health equity and patients’ rights, which are expressed in the Swedish Health and Medical Services Act (SFS 1982:763). Telenurses have expressed dilemmas of being both a gatekeeper to the health care system and a carer for the patient (Holmstrom & Dall'Alba, 2002). This is a dual role which creates tensions. There is a risk that the demands for efficiency and gatekeeping interfere with the goals of equity and good health care. This is also in line with similar analyses of malpractice claimed calls to SHD (Ernesäter, Winblad, Engstrom, & Holmström, 2012).

In our results, the communication between telenurse and caller was also characterized by *a focus on medicine*. The medically underpinned CDSS might contribute to this. The analysis also showed that when the patient used psychosocial talk, the telenurse could steer the communication back to a purely medical type. This happened in call number 4, in which the root of the young girl's stress could not be elaborated within this discourse. The patient expressed *the voice of life world*, whereas the nurse talked in *the voice of medicine*, oriented almost exclusively toward a technical bioscientific understanding of medicine (Mishler, 1984).

According to Mishler (1984) and Waitzkin (1989) this exclusion of social context from critical attention is a defining part of medical ideology, where a biomedical and individualistic understanding of health dominates over critical theories stressing structures and justice (Weber, 2006). Waitzkin (1989, 2011) has pointed out that the medical meeting does not occur in a vacuum; many problems the patient brings to the doctor have societal roots—thus micro-level interactions are shaped by macro-structures of society. Moreover, telenursing is placed in a health care context where nursing is subordinated medicine (Leppänen, 2010), which makes power interactions on many levels important in this type of communication.

Weber & Parrah-Medina (2003) have argued that a narrow medical ideology focuses on the individual's responsibility as the root to illness, rather than social structures, such as discrimination. Based on our analysis, one could argue that the telenursing discourse is even narrower than traditional medical discourse, because several aspects of a traditional medical encounter are absent; the physical examination, the medical history of the caller, questions about co-morbidity, family history, diagnosis, and future planning.

The final theme concerns *gender*. Telenursing has been argued to be a gendered service because a majority of callers as well as telenurses are women, and gender differences in the communication and advice given have been reported earlier (Höglund & Holmström, 2008; Kaminsky et al., 2010). Previous research has identified male callers as either reluctant or assertive (Goode et al., 2004; Seymour-Smith, Wetherell, & Phoenix, 2002). The reluctant behaviour of caller number 1 could be understood as an expression of hegemonic masculinity (Connell & Messerschmidt, 2005) that collides with the ideals of being a patient; such as being weak and able to ask for help (Courtenay, 2000).

Comparing caller 1 (a reluctant male caller) with caller 2 (a young female caller), it can be argued that the woman acts in line with ideal femininity, which goes hand in hand with being a patient (Lyons, 2009), whereas the man does not. Also call number 4 can be understood in the light of gender construction, as gender biases might have been at play when the woman's symptoms were defined as related to stress and being a new mother, although the CDSS urged her to seek the emergency room. We argue, that this begs the question whether the telenurse would have drawn the same conclusions for a new father with the same symptoms.

According to theories of hegemonic masculinity, one could expect the immigrant man in call number 3 to act as an assertive caller, demanding service and showing gendered power. However, his low language proficiency made this power quite weak. Previous studies have indicated that hegemonic masculinity can be beneficial within health care; for example, female callers have reported that they ask their male partners to call if they believe they will not be trusted (Goode et al., 2004; Höglund & Holmström, 2008). In this case, however, the gatekeeping character of the call was seemingly strengthened, and the encounter became almost robot-like, indicating that ethnicity and gender intersected in the communication.

Language proficiency is perhaps even more crucial in telenursing than in other medical encounters, in which you can see the patient and use body language. Although SHD 1177 offers translators, the service still seems to be difficult to use for patients from ethnical minority groups. Primarily elderly male callers with low language proficiency risk being discriminated by the service (Hsu et al., 2011; Knowles et al., 2006).

Following Walzer (1983), one could argue that our results indicate that the discourse of telenursing mirrors how the possession of one type of social good gives access to other social goods, such as health care. This is also in line with theories on intersectionality, according to which different kind of power structures can intersect in people's lives (Weber & Parrah-Medina, 2003).

Call number 5, finally, represents a counter discourse. Here the medical discourse is challenged by the nurse inviting the life world experience of the caller to be part of the consultation. Nursing aspects are part of the call and the caller is instructed about his symptoms. Furthermore, the telenurse does not follow CDSS strictly in this example, which might challenge the knowledge and power asymmetry between telenurse and caller. This example of a counter discourse shows how the caller can be empowered in a telenurse consultation.

The three identified themes (*gatekeeping*, *focus on medicine*, and *doing gender*) can be understood through Fairclough's (1992) three-dimensional framework of discourse, consisting of *the text analysis level*, *the discursive practice level*, and *the social practice level*. The communication in the calls (the text level) was characterized by gatekeeping and a biomedical language. This level stands in dialectical contact with the two other dimensions of discourse: the discursive practice, which concerns how telenursing is performed (tools, goals, organization) and the social practice, which concerns how the dominating ideologies in society stand in contact with telenursing. Following Waitzkin (1989, 2011), the discourse is both socially constitutive and socially conditioned. This means, that the communication in the calls both depends upon and reconstructs the societal discourse.

### Strengths and limitations

The study was qualitative and the results cannot be generalized to a larger population. In addition, the context (primary health care) and culture (Swedish) might have influenced the results. However, we propose that the findings are transferable to similar settings.

In order to achieve trustworthiness the criteria of *credibility*, *dependability*, *confirmability*, and *transferability* as outlined by Guba and Lincoln (1989) were followed. Credibility, that is, to be thorough in data collection and analysis, was respected as the calls were carefully chosen and all authors participated in the analysis process. Dependability, meaning consistency and that the research process should be described so that it is easy to follow, has been fulfilled through the description in the method section. Confirmability refers to the notion that the research should convincingly show how the results are grounded in the material, which has been fulfilled through the use of transcripts of calls. Finally, transferability, meaning that the results can be readily communicated and useful in other contexts, has been fulfilled through the discussion of the results.

Through the CDA, the social context and power distribution in the calls could be addressed. In order to increase the transparency and the validity of the analysis, long extracts from different calls were presented. The analysis showed that language proficiency and gender can affect the communication simultaneously. However, other categories, such as social class, could not be analysed in this material, although it might play an important role in the medical encounter. Through the CDA, a counter discourse was also found, deepening the understanding of the communication and exploring how the interaction between caller and telenurse not only reflects the macro-system, but also can contribute to its transforming.

## Conclusion

The dominating patterns in the studied calls were of gatekeeping and biomedical character. Furthermore, the societal gender order was mirrored in the calls, as representations of the reluctant male caller and the ideal feminine caller were found. The service seemed difficult to use for patients with low language proficiency. Health inequities are one of the largest challenges for all welfare systems. Telenursing could potentially challenge inequalities, because the service is cheap and also easy to access in rural areas. However, the discourse of telenursing is dialectically related to neoliberal ideology and the ideology of medicine. It is also situated in a gendered context of ideal femininity and hegemonic masculinity. Through awareness of gender biases and the callers’ different resources for making themselves heard, the communication between telenurse and caller can be made more equal and thereby better suitable for all callers.
